# Research on Psychache in Suicidal Population: A Bibliometric and Visual Analysis of Papers Published During 1994–2020

**DOI:** 10.3389/fpsyt.2021.727663

**Published:** 2021-08-26

**Authors:** Yin Cheng, Wei-Wei Zhao, Shu-Yan Chen, Yan-Hong Zhang

**Affiliations:** ^1^Department of Nursing, Affiliated Nanjing Brain Hospital, Nanjing Medical University, Nanjing, China; ^2^School of Nursing, Nanjing Medical University, Nanjing, China; ^3^Department of Psychiatry, Affiliated Nanjing Brain Hospital, Nanjing Medical University, Nanjing, China

**Keywords:** psychache, bibliometric analysis, hotspots, Web of Science, CiteSpace, suicide

## Abstract

**Background:** Psychache is a negative introspective experience, which is positively associated with the risk of suicide, independently of depression. It is undeniable that psychache is an important influencing factor to trigger suicide, which can also mediate the effect between depression and suicide variables. Nevertheless, the research tendency and current hotspots on psychache of suicide population have not been systematically investigated based on bibliometric analysis.

**Aim:** The aim of the study was to analyze the research status, hotspots, and frontiers of psychological pain in the field of suicidology, so as to provide reference for domestic clinical research.

**Methods:** The literature related to psychache in suicide individuals published from 1994 to 2020 was included and selected from the Web of Science Core Collection database on May 28, 2021. CiteSpace (version 5.7.R2) software was used to visualize and analyze highly cited journals, authors, and articles as well as co-occurrence analysis for countries, institution, authors, and keywords.

**Results:** A total of 230 articles from the WoS database were included. The number of papers over the years showed an overall upward trend. The United States has made the largest contribution, with the majority of publications (89, 38.70%), followed by Canada (48, 20.87%), Israel (31, 13.48%), China (20, 8.80%), and Portugal (17, 7.39%). The most productive institution was Queen's University. Edwin S. Shneidman has the largest achievement and profound influence, and the most prolific author is Ronald R. Holden. However, the cooperation between institutions and authors was comparatively weak. The current hotspots in this field focus on the studies on the relationships between depression, despair, psychache, and suicide, the risk assessments of psychological pain, and the development of psychological pain scales. *Suicide and Life-Threatening Behavior* was the most frequently cited journal in this field.

**Conclusions:** This analysis not only reveals the current research trend and hotspots but also provides some instructive suggestions on the development of psychache in the suicidology. Future work should pay more attention to develop effective psychological pain intervention programs for diverse suicide population. Additionally, longitudinal study can also be conducted to grasp the trajectory changes of psychological pain among suicide individuals.

## Introduction

According to the Global Health Estimates of Suicide in 2019 from World Health Organization, globally, over 703,000 people die by suicide every year, and more than one in every 100 deaths (1.3%) in 2019 were the result of suicide (1). The suicide death rate in China reached to 64.1% in 2016 (2). Obviously, suicide has already become a grave public health issue. Suicide is regarded as a multifactorial phenomenon, including psychological (e.g., personality traits, dysregulation), biological (e.g., genetics, comorbid illness), and environmental factors (e.g., social support, family) (3). Despite various risk factors have certain influence on the prediction of suicide, in particular, psychache that has been empirically related to suicide is the central role in suicidal behavior. “Unbearable” psychological pain is one of the most common complaints in suicide population (4). In other words, suicide would not occur without psychological pain.

As a complicated multi-dimensional structure, psychache (mental pain and psychological pain) is constituted by physiological, cognitive, action tendency components (5). Even though the definition of psychache is controversial and complex, the theoretical definition provided by Shneidman has been widely accepted by researchers in the field of suicidology. According to Shneidman's theory of suicide, psychache is defined as a passive introspective experience encompassing guilt, shame, humiliation, dread, and loss. Orbach et al. have described nine characteristics of psychological pain, namely, emptiness, loss of control, narcissistic wounds, emotional flooding, freezing, estrangement, confusion, social distancing, and the experience of irreversibility (6). The significant association between psychache and suicide has been ascertained with majority empirical studies in diverse groups (e.g., offenders, students, homelessness, and patients with depression) showing that the level of psychological pain could predict suicidal ideation and action (7, 8). A 2-year longitudinal study by Troister et al. has confirmed that psychache is the sole unique predictor of suicide ideation in a sample of college students (9). Besides, emerging studies further find that suicide is a mean to alleviate an inner painful state for suicidal individuals (10, 11). That is to say, suicide is a problem-solving act suspending the painful experience. In agreement, the motivation to escape from painful self-awareness has been emphasized in Baumeister's escape theory of suicide (12).

Additionally, three models of evoking psychological pain from the perspective of pathophysiology established by Gaillard, namely, sympathy pain, loss pain, and social exclusion pain, show that the specific brain's regions are activated through the paradigms of financial loss, social exclusion, and pain empathy; the result indirectly reflects that psychache may have a harmful effect on the treatment and prognosis of disease (13, 14), especially in patients with depression. A brief self-report scale developed by Mee et al. (15) was used to estimate psychological pain in depressed patients. The result showed that the score was positively correlated with depressive symptomatology (15). From this, psychological pain may be a prominent symptom in people with depression, but this view needs to be confirmed in the future.

As a major hazard contributor of suicidal component (ideation, attempt, and behavior), there is no doubt that psychache is the key to prevent individual suicide. Currently, the overall level of psychache of depressed patients in China is in the above medium degree (16). Given that the grave situation, showing concern for patients' psychache is critical in the implementation process of the mental health action plan of Healthy China 2030 and psychological service, among these segments of implementation, bringing psychache score into the routine admission assessment, combined with the depression score, is of great importance in reinforcing suicide management.

As a novel method of scientometrics, knowledge map is an image that regards knowledge area as a research object and presents the evolution process of scientific knowledge. It is not only a visual knowledge graph but also a serialized intellectual genealogy, with the “graph” and “spectrum” properties (17). CiteSpace, developed by Professor Chen of Drexel University (18), is one of the most prevalent knowledge mapping instruments, which integrates social network analysis, cluster analysis, and multi-dimensional scale analysis as well as focuses on analyzing, mining, and seeking research hotspots and development trends in a certain discipline (19).

With the increasing number of suicide deaths, far too little attention has been paid to the psychological pain in China. This paper provides an overview of hot spots and research trend of psychological pain from the perspective of bibliometrics, for the sake of offering evidence for the development of domestic research.

## Materials and Methods

### Data Collection and Search Strategy

The bibliometric data were drawn from the Web of Science Core Collection (WoSCC) database from January 1, 1994 to December 31, 2020. The search equation was TS = [(mental pain OR psychological pain OR emotional pain OR psychache OR psychic pain) AND (suicide^*^)]. The publication type was limited to “Article.” All records and citations were imported into Endnote X9 software and saved as plain text.

### Inclusion and Exclusion Criteria

The inclusion criteria were as follows: (1) The journal articles related to “suicide” and “psychache”; (2) Published between January 1, 1994 and December 31, 2020; and (3) Published in English.

The exclusion criteria were as follows: (1) Did not use English as publication language; (2) The citations were “Correction,” “Case Report,” “Commentary,” “Letter,” “Abstract,” “Reply” instead of journal articles; (3) Repetitive publication; (4) The article is without abstract; and (5) The topic only involved psychache or suicide.

### Data Analysis

The files downloaded from the WoS database were saved as plain text, then the CiteSpace V (5.7.R2) Software (https://sourceforge.net/projects/citespace/) was used for construction and visualization of network (20). The specific parameters used in CiteSpace were set as follows: time span (from January 1, 1994 to December 31, 2020, year per slice = 1), top *N* (50), term source (title, abstract, author keywords, and keywords plus), node types (author, institution, country, keywords, cited reference, cited author, and cited journal), network clipping method (pathfinder) (21). In this study, log-likelihood ratio (LLR) was employed to cluster keywords; the clusters with the silhouette value ranging from 0.5 to 1.0 were selected (22).

## Results

### Distribution Characteristics of Publications

**Figure 1:**
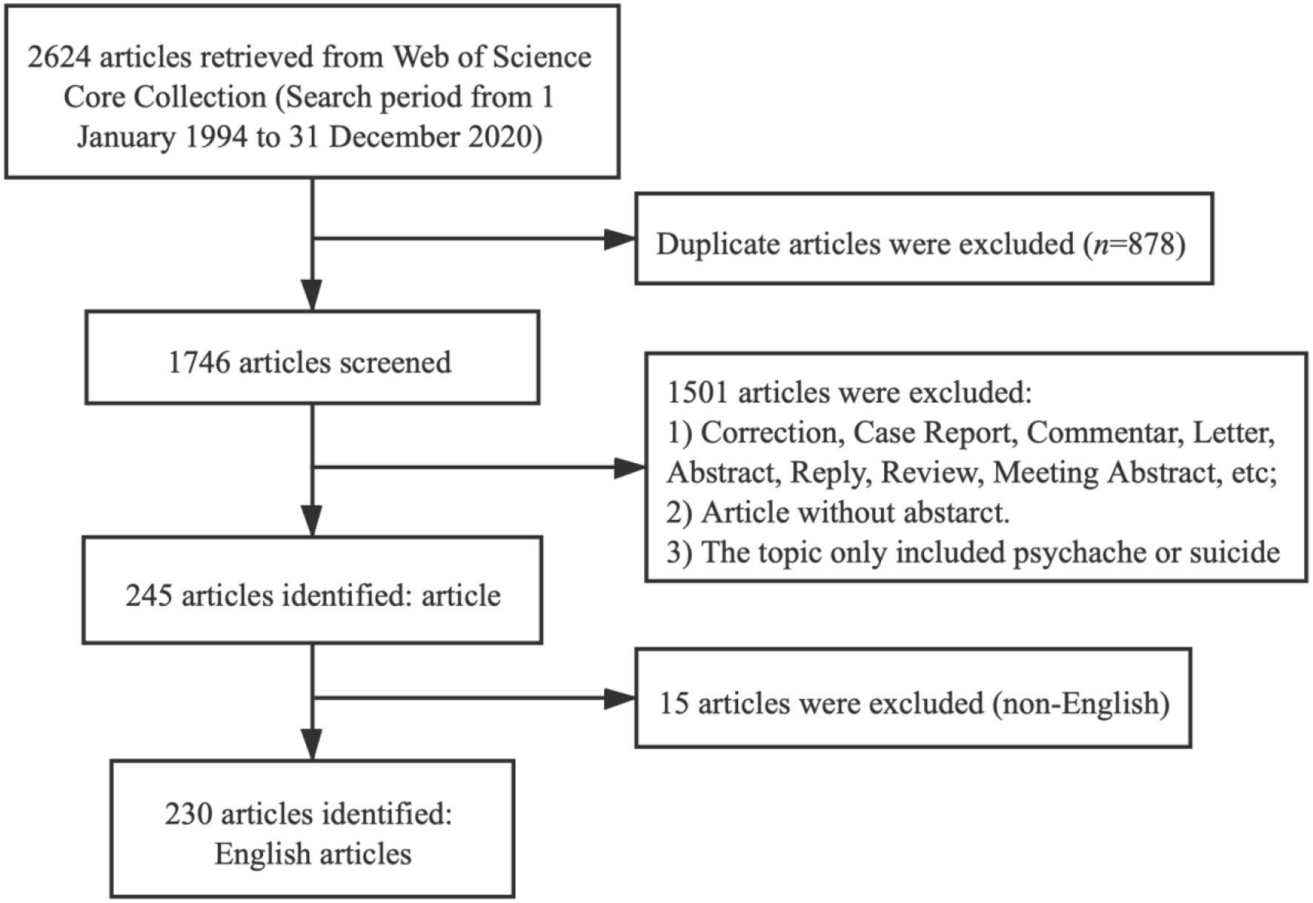
The flowchart of included and excluded studies.

**Figure 2:**
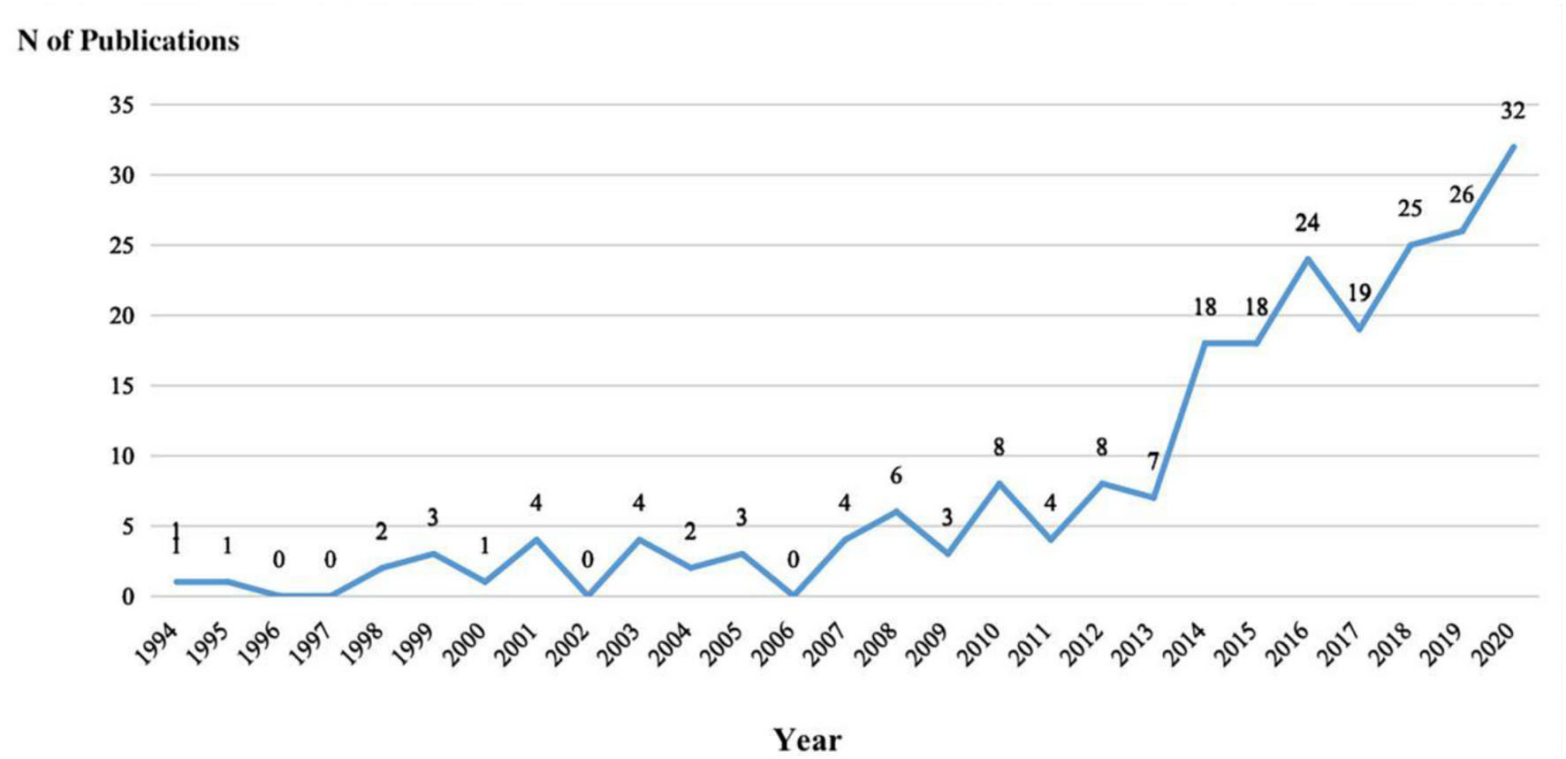
Trend in the number of publications on psychache in suicidology included in WoS database from 1994 to 2020.

In all, 2,624 citations were primarily searched. After removing 878 duplicates, the remaining 1,746 articles were further assessed, of which 230 articles were eventually processed by CiteSpace V (5.7.R2) Software. Figure 1 shows the flowchart of the included and excluded studies. The global research on psychache in the field of suicidology during the period of 1994–2020 is shown in Figure 2. In spite the annual publications were at low level, the largest count only reached to 32, it still presented an overall upward trend. However, the studies on psychache in the field of suicidology had a very slow development speed between 1994 and 2013. After 2013, the publication output had a rapid development period, in which production significantly increased by 75%, the phenomenon indicated that more attention has been given to psychache in the past decade. In the coming years, the research tendency will continue to be great.

### Analysis of Scientific Collaboration Network

**Figure 3:**
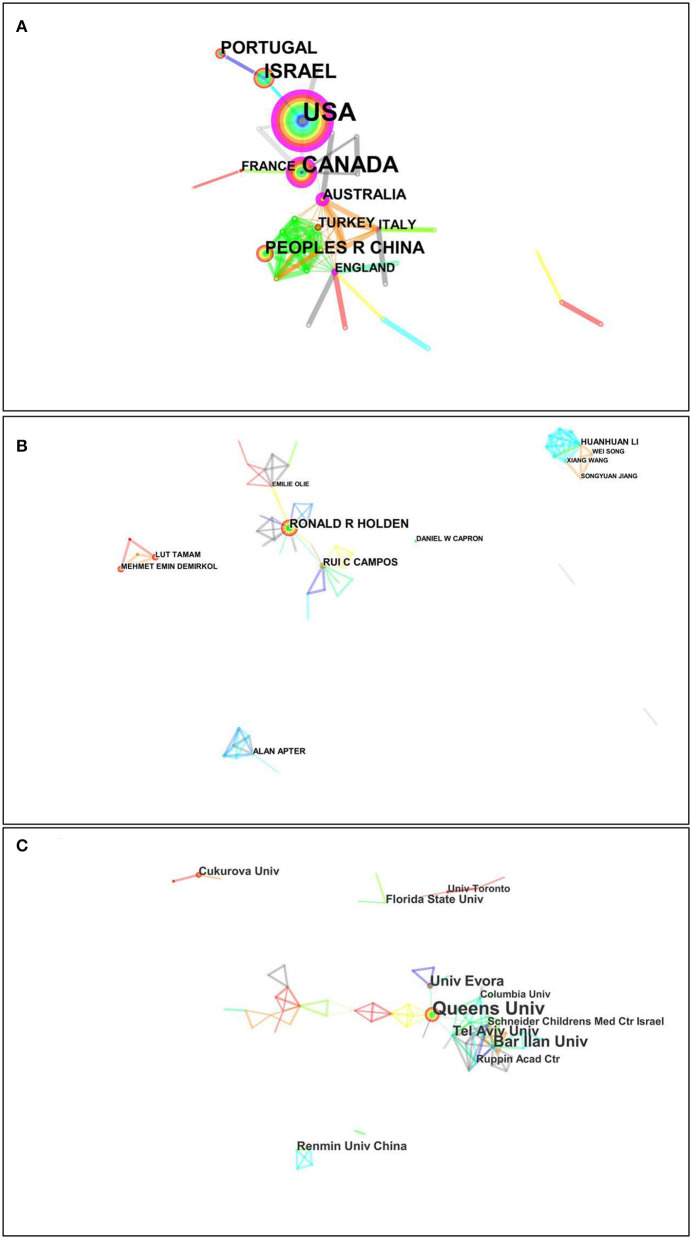
Visualization map of the scientific collaboration network analysis of psychache research in the suicidology field from 1994 to 2020. Collaboration among countries **(A)**, authors **(B)**, and institutions **(C)**.

The analysis of distribution of countries, institutions, and authors is to understand the current international scientific research center of psychache in suicide population. The top 10 countries, institutions, and authors in terms of publication outputs during 1994–2020 are listed in Table 1. Among these countries/regions, the United States was the country with the highest production (publications: 89), accounting for 38.70% of the total number of papers published, followed by Canada (publications: 48, 20.87%), Israel (publications: 31, 13.48%), China (publications: 20, 8.70%), and Portugal (publications: 17, 7.39%). At present, the research on suicide-related psychache mainly focuses on the North America, Europe, Asia, and the Middle East, of which the United States possesses prominent strength in this field. The visualization map of the scientific collaboration network among countries is presented in Figure 3A, in which there were 44 nodes and 86 link lines (density: 0.909). The size of the node represents the amounts of papers, the larger the node, the more publication. The connection indicates a cooperative relationship between countries/regions, the thickness of connection indicates the cooperation intensity; the thicker the line, the closer the relationship. Among these countries, Israel had the largest collaborative connection with other countries/regions (collaborators: 14). Next is China (collaborators: 10), the USA (collaborators: 9), Canada (collaborators: 9) and Portugal (collaborators: 3). Overall, the partner relationships between countries/regions were weak, which attributed to the immature development of psychache in the field of suicidology.

**Table 1:** The top 10 countries, institutions and authors in terms of publications included in WoS database during 1994–2020.

**Country**	**Publications, *n* (%)**	**Institution**	**Publications, *n* (%)**	**Author**	**Publications, *n* (%)**
USA	89 (38.70)	Queen's University	30 (12.88)	Ronald R. Holden	25 (10.87)
Canada	48 (20.87)	Bar-Ilan University	16 (6.87)	Rui C. Campos	13 (5.65)
Israel	31 (13.48)	University of Evora	13 (5.58)	Huanhuan Li	8 (3.48)
China	20 (8.70)	Tel-Aviv University	12 (5.15)	Alan Apter	8 (3.48)
Portugal	17 (7.39)	Renmin University of China	8 (3.43)	Mehmet Emin Demirkol	6 (2.61)
Australia	12 (5.22)	Florida State University	7 (3.00)	Lut Tamam	6 (2.61)
Turkey	10 (4.35)	Cukurova University	7 (3.00)	Daniel W. Capron	5 (2.17)
France	8 (3.48)	Schneider Children's Medical Center of Israel	6 (2.58)	Songyuan Jiang	4 (1.74)
Italy	8 (3.48)	Ruppin Academic Center	6 (2.58)	Xiang Wang	4 (1.74)
England	7 (3.04)	University of Toronto	5 (2.15)	Wei Song	4 (1.74)

In the ranking of scientific research institutions, Queen's University had the largest number of papers (publications: 30), accounting for 12.88% of the total number of publications, followed by Bar-Ilan University (publications: 16, 6.87%), University of Evora (publications: 13, 5.58%), Tel-Aviv University (publications: 12, 5.15%), and Renmin University of China (publications: 8, 3.43%), as shown in Table 1. Majority institutions in this field came from the Department of Psychology. Figures 3B,C unfold the visualization map of the scientific collaboration network among authors and institutions. Bar-Ilan University (collaborators: 18) embraced the widest cooperative relationship, followed by Tel-Aviv University (collaborators: 15), Queen's University (collaborators: 8), University of Evora (collaborators: 4), and Renmin University of China (collaborators: 3).

### Analysis of Co-occurring Keywords

Keyword frequency analysis is an analytical approach to capture research hotspots, knowledge structure, and development trend in a subject (24). This analysis method is of great significance for mastering the research trend. It can be seen from Table 2 that in terms of co-occurrence frequency, the top 10 keywords were “Depression,” “Suicide,” “Scale,” “Risk,” “Behavior,” “Ideation,” “Hopelessness,” “Meal pain,” “Psychache,” and “Mental health.” According to the above keywords, the research hotspots of suicide-related psychache can be summarized into three aspects: (1) the relationship between depression, despair, psychache, and suicidal behavior/ideation, (2) risk assessment of suicide, and (3) the study on psychache scale.

**Table 2:** The top 10 keywords in terms of frequency for psychache in the suicidology field.

**Ranking**	**Keywords**	**Betweenness centrality**	**Frequency**
1	Depression	0.13	102
2	Suicide	0.03	66
3	Scale	0.02	50
4	Risk	0.11	47
5	Behavior	0.11	47
6	Ideation	0.10	44
7	Hopelessness	0.27	39
8	Mental pain	0.06	39
9	Psychache	0.06	37
10	Mental health	0.20	34

### Analysis of High-Cited Journals, Literature, and Authors

Mutual citation and co-citation cannot only reflect the connection between documents and disciplines but also obtain the knowledge of various disciplines (22). Table 3 presents the top 10 highly cited references for psychache in the field of suicidology. The article published by Maria Cristina Verrocchio in *Frontiers in Psychiatry* was the most highly cited reference (23). It is noteworthy that this article is the first systematic review to comprehensively illustrate the association between suicide attempt/behavior and psychache based on diverse groups (e.g., patients with affective disorder, students, homelessness, and soldiers). Additionally, this article provides an important reference for the design of psychache intervention scheme and the exploration of influencing factors on psychache.

**Table 3:** The top 10 journals, authors, and articles in terms of citation frequency during 1994–2020.

**High cited journal**			**High cited author (citation frequency)**	**High cited article**				
**Journal**	**Impact factor**	**Citation frequency**		**Title**	**Author**	**Journal**	**Year**	**Citation frequency**
Suicide and Life- Threatening Behavior	3.867	168	Aaron T. Beck (92)	Mental pain and suicide: a systematic review of the literature	Maria Cristina Verrocchio	Frontiers in Psychiatry	2016	27
Journal of Affective Disorders	4.839	120	Edwin S. Shneidman (88)	A two-year prospective study of psychache and its relationship to suicidality among high-risk undergraduates	Talia Troister	Journal of Clinical Psychology	2012	26
American Journal of Psychiatry	18.112	103	Ronal R. Holden (68)	Comparing psychache, depression, and hopelessness in their associations with suicidality: a test of Shneidman's theory of suicide	Talia Troister	Personality and Individual Differences	2010	22
Journal of Consulting and Clinical Psychology	5.348	96	Isael Orbach (55)	Psychache and suicide ideation among men who are homeless: a test of Shneidman's model	Allisha A. Patterson	Suicide and Life-Threatening Behavior	2012	21
Journal of Nervous and Mental Disease	2.254	92	Talia Troister (50)	Suicide risk screening: comparing the beck depression inventory-II, Beck hopelessness scale, and psychache scale in undergraduates	Talia Troister	Psychological Assessment	2015	18
Journal of Clinical Psychology	2.885	88	Ronal C. Kessler (48)	Differentiating between depression, hopelessness, and psychache in university undergraduates	Michelle M. DeLisle	Measurement and Evaluation in Counseling and Development	2009	17
Psychological Medicine	7.723	84	Matthew K. Nock (35)	Factorial differentiation among depression, hopelessness, and psychache in statistically predicting suicidality	Talia Troister	Measurement and Evaluation in Counseling and Development	2013	17
Archives of General Psychiatry	14.480	82	American Psychiatric Association (33)	Diagnostic and statistical manual of mental disorders (5th version)	American Psychiatric Association.	Washington, DC: American Psychiatric Association	2013	16
British Journal of Psychiatry	9.319	78	Roy F. Baumeister (32)	Clarifying the role of psychological pain in the risks of suicidal ideation and suicidal acts among patients with major depressive episodes	Huanhuan Li	Suicide and Life- Threatening Behavior	2014	15
Canadian Journal of Behavioral Science- Revue Canadienne des Science du Comportement	0.273	71	Augustine Osman (30)	Preventing suicide: a global imperative	World Health Organization	https://www.who.int/mental_health/suicide-prevention/world_report_2014/en/	2014	15

Table 3 presents the top 10 highly cited journals for psychache research in the field of suicidology. Among the top three highly cited journals, the most influential journal in this field is *Suicide and Life-Threatening Behavior* (IF = 3.867), which is created by the American Association of Suicidology (AAC), followed by *Journal of Affective Disorders* (IF = 4.839) and *American Journal of Psychiatry* (IF = 18.112).

The top five high-cited authors are from the United States, Canada, and Israel, as shown in Table 3. Of these authors, Edwin S. Shneidman, the founder of AAS, is an authority in the field of suicidology. He first put forward the concept of “psychache” in the early 1990s and considered that psychache was the leading cause of suicide. Subsequently, Ronald R. Holden and Talia Troister et al. in the Department of Psychology of Queen's University as well as Israel Orbach et al. in the Department of Psychology of Bar-Ilan University executed the research on suicide-related psychache in the early twenty-first century, and now they have become the force at the core in the field of suicide-related psychache.

### Analysis of Keywords Clusters

**Figure 4:**
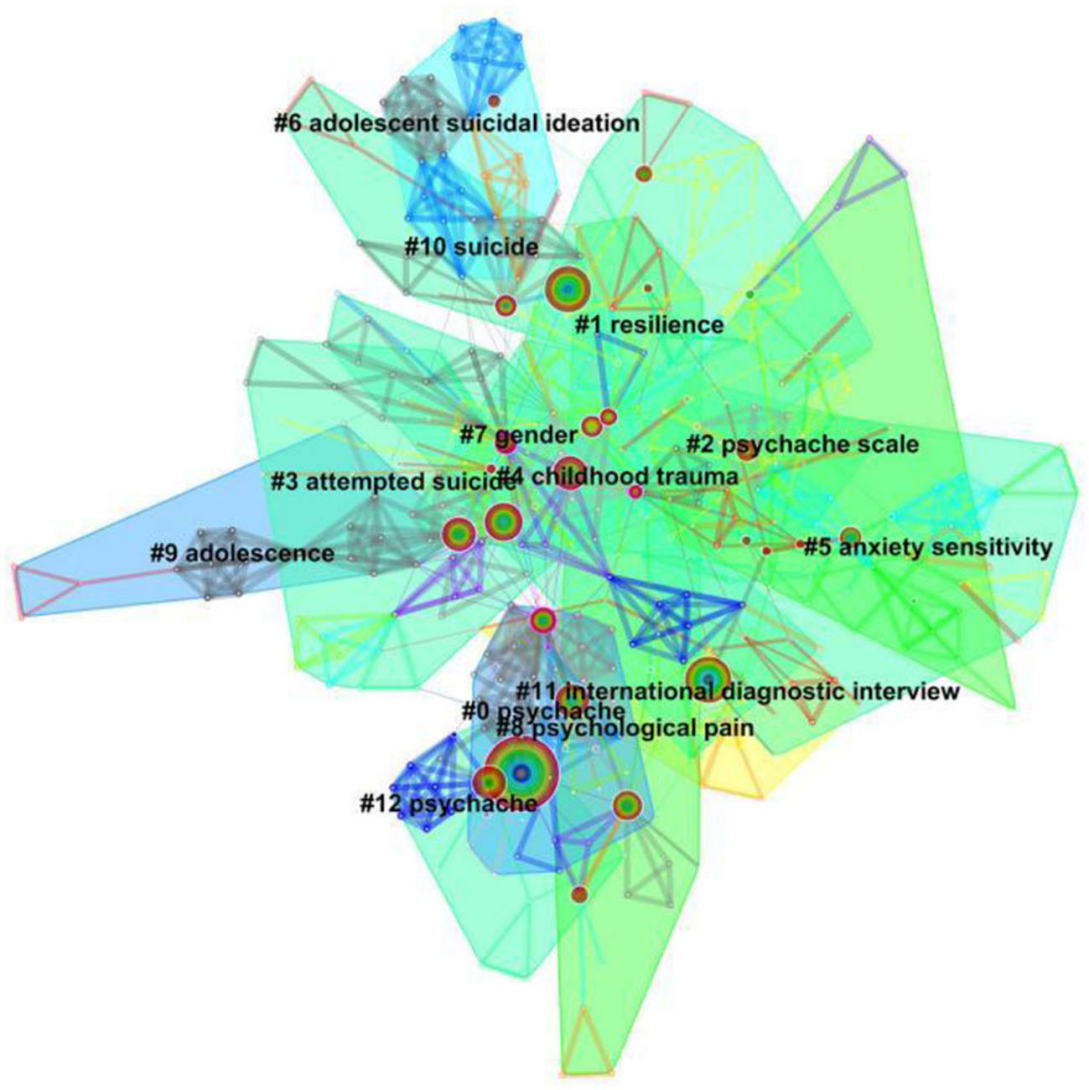
Cluster visualization of the keywords co-citation map.

The LLR method was utilized to cluster keywords in the present study. A total of 13 keyword clusters were formed. The visualization map of keyword clusters is shown in Figure 4. Researchers selected the first seven clusters and extracted the main keywords. The silhouette values of cluster 0–12 were in the range of 0.827–0.992, which showed the good homogeneity, as shown in Table 4. The largest visual cluster was #0 psychache, followed by #1 resilience, #2 psychache scale, #3 attempted suicide, #4 childhood trauma, #5 anxiety sensitivity, and #6 adolescent suicidal ideation. The research topics can be mainly summarized into three respects: (1) influencing factors on psychache in suicide populations with different age and background, (2) reliability and validity of psychache scales, and (3) the model construction of the relationship between psychache and suicide variables.

**Table 4:** The top 7 clusters of keywords for psychache in the suicidology during 1994–2020.

**Cluster ID**	**Major keywords**	**Content and interpretation of the cluster**	**Size**	**Silhouette value**	**Mean (year)**
#0	Depression, hopelessness, psychache, eventual suicide, perfectionism,	The model construction of suicide and psychache.	38	0.927	2006
#1	Anxiety, social support, substance abuse, self esteem, adjustment,	Positive mental change in suicidal population.	37	0.883	2014
#2	Validity, prevention, psychache scale, stress, symptom, suicide ideation	Psychometric test of psychache.	37	0.845	2013
#3	Association, attitude, death, loneliness, severity, bereavement, cognition?impact	Application study of psychache on bereaved.	35	0.924	2013
#4	Behavior, disorder, suicide attempt, children, maltreatment, abuse, adverse	Application study of psychache on children suicide population.	32	0.868	2010
					
#5	Risk factor, college student, comorbidity, suicidality, survivor, anxiety	Application study of psychache on college students and survivors.	27	0.91	2014
					
#6	Youth, post-traumatic stress disorder, gender difference, intimate partner violence	Application study of psychache on adolescent suicide population.	27	0.986	2011

### Analysis of Burst Detection

**Figure 5:**
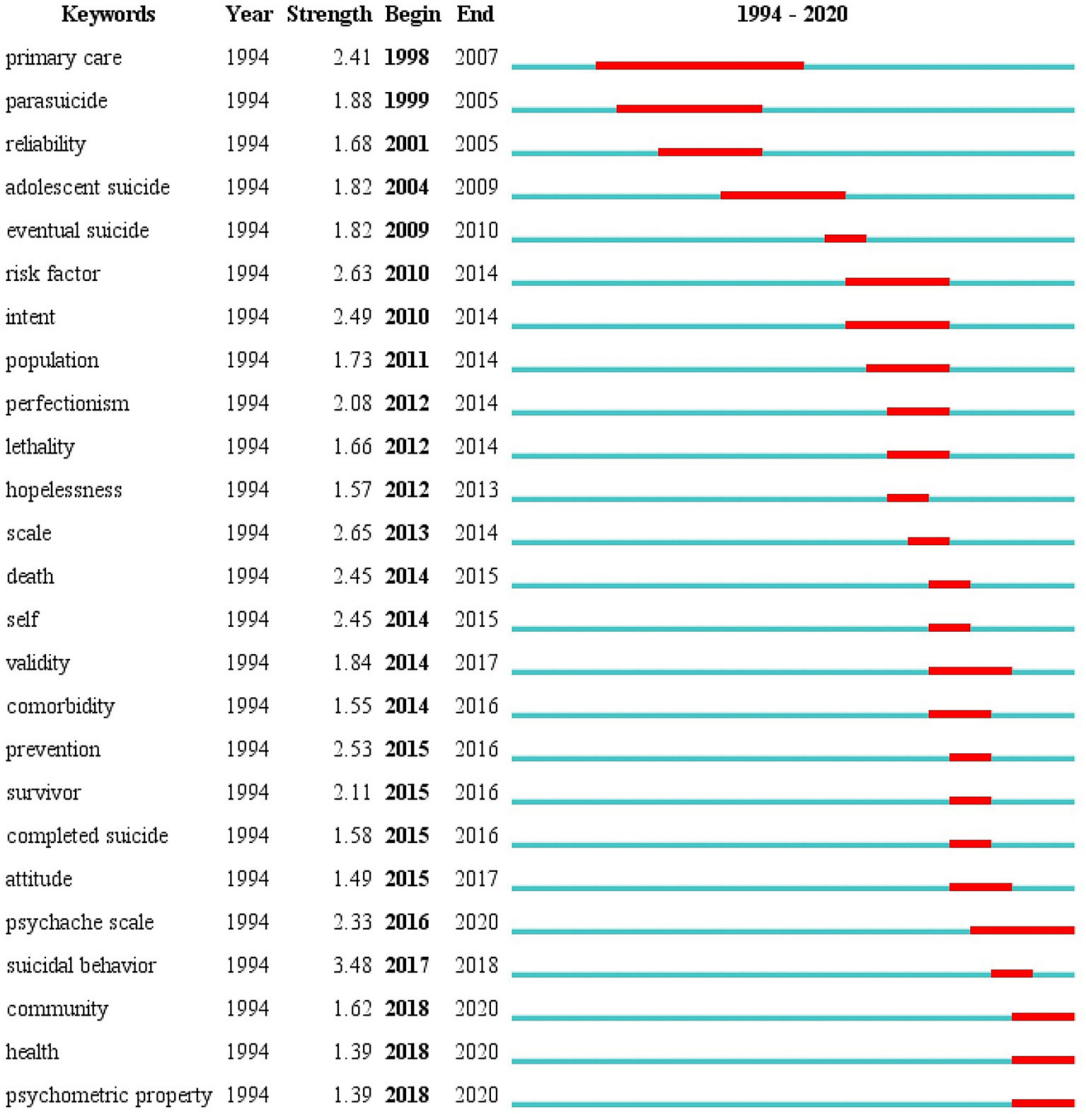
The 25 burst terms for psychache research in the suicidology field from 1994 to 2020.

Burst keywords are detected from numerous subject terms and determine the new research topics of a research field, according to the change trend of word frequency. The term with high burst intensity (burst value) represents the frontier of this stage (25). Figure 5 shows the top 25 keywords with the strongest citation bursts. “suicidal behavior” was the strongest burst keywords (intensity: 3.48) from 2017 to 2018, followed by “scale (intensity: 2.65),” “risk factor (intensity: 2.63),” and “prevention (intensity: 2.53).” The keywords with a burst lasting until 2020 included “psychache scale (intensity: 2.33),” “community (intensity: 1.62),” “health (intensity: 1.39),” and “psychometric property (intensity: 1.63).”

**Figure 6:**
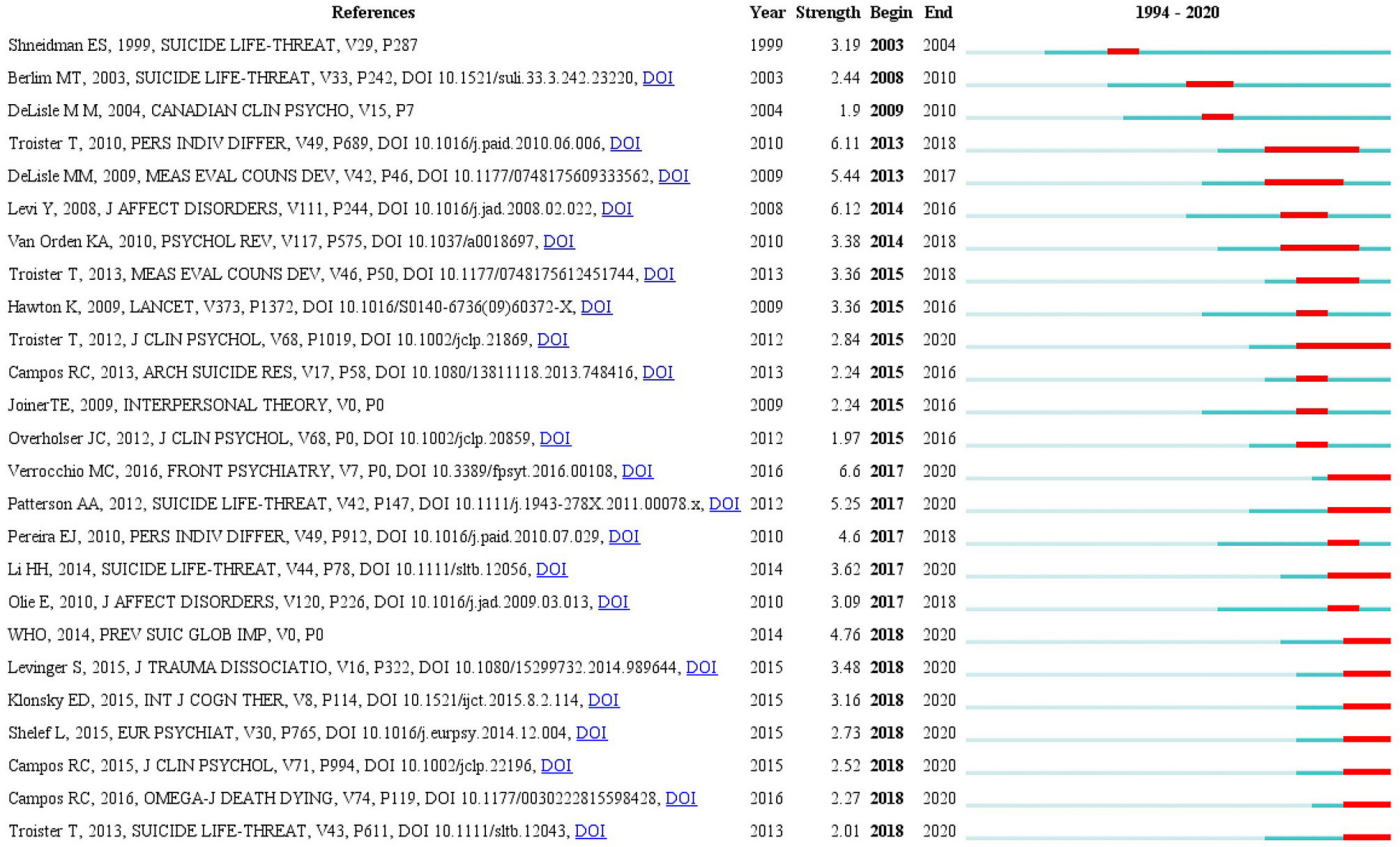
The 25 burst references related to psychache research in the suicidology field between 1994 and 2020.

References with citation burst refer to those that suddenly have high citation frequency. These references can be used to reflect new themes and important research directions (26). A total of 25 references with strong citation bursts were detected (Figure 6). The most recent references with citation bursts appeared in 2018. The article with strongest burst (intensity: 6.12) appeared in 2014, which was published by Levi et al. in the *Journal of Affective Disorders* (27). Seven references had a burst that lasted until 2020 (28–34).

## Discussion

This is the first application of bibliometric and visual analysis methods to psychache research in the field of suicidology. In all, 230 articles originated from WoS Collection Core database were analyzed. By analyzing the author co-occurrence network map and the ranking of high-cited authors over the past two decades, two research teams from Queen's University and Bar-Ilan University as the core were formed. In the present study, Ronald R. Holden from the department of psychology of Queen's University was the most prolific author in this field. The international studies on psychache mainly concentrated on the development of psychache scales, investigation of the relationships between psychache and suicide in a variety of groups, as well as the mediating effect of psychache in suicide path. In turn, studies on psychache within China focused on the behavior and event-related potential (ERP) research of suicide mechanism grounded in the psychological pain theoretical models. The relationship between suicide and psychache in Chinese college students was another main research interest presently undertaken by Caizhi Wu et al. who come from the School of Psychology of Central China Normal University.

Most of the highly cited journals were published by America. As the most powerful journal in the field of suicidology, *Suicide and Life-Threatening Behavior*, founded by AAS, is terribly important for researchers who engage in the field of psychache. Paying more attention on high-cited journals and authors is an accessible approach to comprehend the influential journals and research groups, which can help researchers grasp the latest research trend and provide valuable guidance for research direction.

The analysis of research topics revealed that most studies explored the risk factors affecting psychological pain only from a macro perspective. Despite mounting evidence showing that psychache is affected by cognition (16), personality traits (35), family (36), etc., it is necessary to make numerous in-depth studies on the mechanism of psychache in view of the structure complexity and prediction ability to suicide of psycache. Recently, the evidence of non-invasive functional brain imaging of psychache has gradually accumulated many results of neuroimaging studies have reported the significant associations between psychache and the cerebral perfusion changes of emotion regulation center and the complexity of left frontal lobe (37, 38). For instance, Reisch et al. found that the activation of orbitofrontal cortex and anterior cingulate gyrus was obviously abnormal in depressed patients with suicide attempts (39). However, due to the limitations of research method and the lack of multi-level integration with suicide theories, it is hard to provide conclusive explanations for the above findings. Future work is required to establish the available experimental paradigms corresponding to suicide theories, in order to further elaborate the physiological mechanism of psychological pain among suicide individuals.

The model construction of psychache and suicide was the largest visual cluster in the current study. The ultimate target of the model construction is to improve the current status of suicide through a range of valid and focal interventions. Even though well-being therapy and expressive writing are considered to be able to alleviate painful experience (40, 41), the explicit intervention programs and large-sample randomized clinical trials (RCT) have not been designed. Zou et al. performed an RCT in 19 Chinese depressed outpatients for an 8-week intervention with the psychological pain theory-based cognitive therapy (PPTBCT) (42), while the result showed an unsatisfied long-term therapeutic effect of PPTBCT on patients with major depressive episode. Thus far, the formulation of psychache intervention projects is still in its infancy. The possible explanations for this might be that the intervention populations are comparatively simplistic, mainly the hospitalized patients with suicidal ideation. Additionally, as a chronic negative emotional experience and psychological manifestation, patients' mood fluctuation has not been constantly followed up and dynamically monitored after discharge. In this regard, research sites should not only be in the clinical setting but also in basic medical and health institutions and schools. To strengthen the interconnection between communities and tertiary hospitals, a hospital–community joint rehabilitation model should be built. There is, therefore, a definite need for establishing a multidisciplinary team, including psychiatric nurses, psychiatrists, psychological counselors, general practitioners (GP), and community nurses, in order to urgently and effectively adjust treatment plans for patients.

Considering the results of high-frequency keywords and burst term detection, this study found that psychache assessment tool was a current research hotspot and frontier in the field of suicidology. Undeniably, an available psychache instrument is the core of suicide prevention. So far, multiple psychache measures have been compiled and applied in Canada, China, France, Israel, including the Psychache Scale (PAS) (43), the Three-Dimensional Psychological Pain Scale (TDPPS) (44), the Psychological Pain Visual Analog Scale (PP-VAS) (45), the Orbach and Mikulincer Mental Pain scale (OMMP) (6), Unbearable Psychache Scale (UP3) (46), the Tolerance for Mental Pain Scale (TMPS) (47), etc. Of these scales, PAS is the most widely used and oft-cited measures of psychache, which was designed by Holden et al. in 2001. This scale has been translated into Chinese by Yang et al. and utilized in Chinese university students, with good reliability and validity (48). Referring to the 13-item PAS, three-item Unbearable Psychache Scale was developed from items 10, 11, and 12 of the PAS, which also demonstrated excellent internal reliability, strong convergent and predictive validity in psychiatric inpatient adults and general adults (46). In addition, TMPS is the first instrument to assess tolerance for mental pain, which is composed of three aspects: (a) ability to tolerate pain surfeit, (b) belief in the ability to cope with pain, and (c) containing the pain (47). Meerwijk et al. further simplified the TMPS, forming the 10-item TMPS. TMPS-10 contains two dimensions: (a) managing the pain and (b) enduring the pain. Cronbach's alpha coefficient was 0.84 for enduring the pain and 0.90 for managing the pain, which showed good internal consistency (49).

Due to majority of the psychache scales introduced from abroad, there are some matters in the process of cross-culture adaptation and application. On the one hand, the equivalence of semantic, content, and concept may be difficult to be guaranteed because of the culture discrepancies between China and the West. On the other hand, majority scales are prone to be self-reported, with subjective items, single dimension, and low specificity. Hence, in terms of the accuracy of evaluation, greater efforts are needed to develop objective scales from the perspective of a second party (e.g., caregivers, families, and medical professionals). However, the challenge now is to enhance predictive validity, sensitivity, and specificity of scales, so it is necessary to conduct large-sample, multicenter psychometric tests.

At the early stage, despair, depression, and loneliness were the main contributors inducing the occurrence of suicide (behavior, idea, or attempt) (50). Since the proposition of Shneidman's trigger theory of psychological pain, data from cross-sectional and longitudinal studies have identified that psychological pain is more predictive of suicide than hopelessness and depression both in non-clinical groups (college students, soldiers, and criminals) (7, 30, 51) and clinical people with depression (15, 52). Wu et al. provides an empirical support for the finding that psychache plays a mediating role in the prediction of depression and despair on suicidal ideation (53). Furthermore, the three-dimensional psychological pain model constructed by Li et al. includes pain avoidance, pain experience, and pain arousal factors, of which pain avoidance has the highest predictive validity for suicide behavior in patients with depression. Based on this model, research indicates the emphatic effect of a motivation ingredient of pain avoidance on triggering suicide (44).

Psychache is a core clinical element for understanding suicide, both in the patients with depression and non-depression. A cross-sectional study by Demirkol et al. (54) demonstrated that psychache might be positively associated with suicide attempts and occurrent suicidal thoughts, and alexithymia might be related to the negative symptom of schizophrenia and psychache in patients with a diagnosis of schizophrenia. Similarly, among the patients with obsessive–compulsive disorder (OCD), a strong correlation was found between psychache and previous suicide attempt (55). Psychache has also been shown to mediate the relationships between suicidal factors and other variables. In a study conducted by Ugur et al. (56), it was shown that psychache had a full mediator role on the effect of sleep disturbance on suicide attempts. The full mediating effect of psychache can also be found on the relationship between childhood trauma and suicide attempts (57).

The findings of the present study provide the following insights for further research directions: (a) the development of objective assessment tools, (b) the exploration of the interaction effect of protective factors (e.g., self-compassion and life satisfaction), psychache, and suicide factors from the perspective of positive psychology, (c) the construction and implementation of intervention plans tailored to diverse population, (d) the development of biomedical and psychological experiments related to psychache, and (e) the conduction of longitudinal studies regarding psychache and analysis of the trajectory changes of psychache in suicide individuals.

The generalizability of the above results is subject to certain limitations. First, this study included documents only retrieved from the Science Core Collection database. Because of the incomplete literature collection, it is most likely that the findings from this analysis may not reflect the actual research trend. In addition, the type of literature was limited to “Article” and references were published only in English, which may make the references included in the current study incomplete, so the results of this study need to be interpreted with caution.

## Conclusion

Through bibliometric analysis and visualization of the included literature, this study has shown that research on psychache in suicidology is developing rapidly at present. The USA is a major producing country, and Ronald R. Holden is a productive author in this field. *Suicide and Life-Threatening Behavior* has the largest publications with regard to psychache. Several future directions and hotspots have been mentioned above, and considerably, more work will need to be done to design and conduct effective interventions among suicide populations so as to decrease the suicide rate.

## Data Availability Statement

The original contributions presented in the study are included in the article/supplementary material, further inquiries can be directed to the corresponding author.

## Author Contributions

YHZ was responsible for the manuscript revision and study design. YC was responsible for the data analysis and writing. SYC and WWZ were responsible for the data analysis and literature review. All authors contributed to the article and approved the submitted version.

## Conflict of Interest

The authors declare that the research was conducted in the absence of any commercial or financial relationships that could be construed as a potential conflict of interest.

## Publisher's Note

All claims expressed in this article are solely those of the authors and do not necessarily represent those of their affiliated organizations, or those of the publisher, the editors and the reviewers. Any product that may be evaluated in this article, or claim that may be made by its manufacturer, is not guaranteed or endorsed by the publisher.
